# Ethanol ingestion via frugivory in wild chimpanzees

**DOI:** 10.1126/sciadv.adw1665

**Published:** 2025-09-17

**Authors:** Aleksey Maro, Aaron A. Sandel, Bi Z. A. Blaiore, Roman M. Wittig, John C. Mitani, Robert Dudley

**Affiliations:** ^1^ Department of Integrative Biology, University of California, Berkeley, CA, USA.; ^2^ Museum of Vertebrate Zoology, University of California, Berkeley, CA, USA.; ^3^ Department of Anthropology, University of Texas, Austin, TX, USA.; ^4^ Systématique et Biodiversité Végétale, Université Félix Houphouët Boigny, Abidjan, Côte d’Ivoire.; ^5^ Taï Chimpanzee Project, CSRS, Abidjan, Côte d’Ivoire.; ^6^ Institute for Cognitive Sciences, CNRS UMR 5229 University of Lyon 1, Bron, France.; ^7^ Department of Anthropology, University of Michigan, Ann Arbor, MI, USA.; ^8^ Smithsonian Tropical Research Institute, Republic of Panama.

## Abstract

Human attraction to alcohol may derive from an evolutionary association between ethanol and fruits consumed by animals in nature. Fermentative yeasts are widespread in the terrestrial biosphere, and simple carbohydrates underpinning ethanol production are commonplace within fruits. We determined ethanol concentrations within fruits representing a substantial portion of the diet of our closest living relatives, the chimpanzees. Ripe fruit pulp from 20 angiosperm species in Côte d’Ivoire and Uganda contained an average value of 0.31 (± 0.21 SD) and 0.32% (± 0.20) ethanol (weight/weight), respectively, as scaled by annual chimpanzee feeding time per species at each site. Chimpanzees typically eat ~4.5 kilograms of fruit per day, corresponding to an estimated ethanol ingestion of 14 grams (±9), or the equivalent of 1.4 (±0.9) standard drinks by international standards. These findings are consistent with the hypothesis that ethanol is widespread within tropical fruits and that modern predisposition to alcohol consumption derives from ancestral exposure to this psychoactive substance among frugivorous primates.

## Teaser

Natural daily consumption of dietary alcohol by chimpanzees suggests human attraction to alcohol may come from our ancestral diet.

## INTRODUCTION

Habitual use of alcohol by modern humans is common around the world. The earliest archeological evidence for controlled fermentation dates to 9000 to 13,000 years ago in China and in the Middle East (*1*, *2*). Intentionally directed fermentation, as a form of predigestion for enhanced caloric extraction, is likely much older (*3*, *4*). Nevertheless, humanity’s attraction to alcohol, both ancient and present, may have deeper evolutionary roots given exposure of our frugivorous ancestors to ethanolic fermentation (*5*). Ethanol, used here synonymously with alcohol, has been hypothesized to be commonplace within the primarily frugivorous diet of our Afrotropical hominid ancestors, resulting in evolution of adaptive physiological responses to its dietary consumption, i.e., the “drunken monkey” hypothesis (*6*). For example, ethanol may function as an olfactory and gustatory indicator of caloric status associated with consumption of ripe fruit, a sensory modality that offers supplemental benefits to any animal that utilizes sugars as part of its primary or secondary diet (*7*). To begin to test these hypotheses, which call for a broader understanding of the evolutionary ecology of ethanol (*8*), describing exposure of chimpanzees (*Pan troglodytes*) to dietary ethanol in their natural habitat is of particular interest. Chimpanzees are one of our two closest living relatives and, along with nearly all extant apes, chronically consume large volumes of ripe fruit. This frugivorous diet is thought to be similar to the diet of our last common ancestor with chimpanzees (*9*, *10*), although it has been suggested that the chimpanzee lineage has continued to evolve toward higher ripe fruit specialization (*11*). Characterizing ethanol concentrations of these fruits and estimating the daily ingested volume of ethanol may accordingly provide insights into its availability to early hominins.

Ethanol is the primary product of anaerobic catabolism of sugars by microbes, typically yeasts. Ethanol production in the yeast family Saccharomycetaceae, to which *Saccharomyces cerevisiae* or brewer’s yeast belongs, has been under natural selection for over 100 million years (*12*, *13*). In these Crabtree-positive yeasts, ethanol is produced via fermentation even in the presence of oxygen, which provides yeast only one-ninth of the energy yield that would otherwise come from oxidative metabolism (*14*). This trait may have evolved as part of a “make-accumulate-consume” strategy, wherein accumulated ethanol acts as a microbicide to initially enhance competition in sugary substrates, followed by reproductive growth as yeasts metabolize the ethanol (*15*). A sugar-rich and progressively more anoxic environment thus facilitates ethanolic fermentation, whereby both facultative and obligate anaerobes compete with one another. Adaptations facilitating these strategies arose concurrently with the worldwide radiation of angiosperms >100 million years ago (Ma) (*16*) and thus coincided with the evolution of fleshy fruits and associated seed dispersal by frugivores (*17*). These adaptations may have evolved as part of a larger mutualistic web (*8*), wherein yeasts receive calories and substrate from the angiosperm in exchange for providing a suite of ecological services to seed dispersers. By biochemically modifying the fruit niche and producing ethanol, yeasts offer a potential long-range signal of calories via ethanol plumes (*7*), an indication of individual fruit quality via olfaction (*18*) and stimulation of appetite during ingestion (*19*). Fermentative yeasts may thus play an ecological “silent third partner” mutualistic role for seed dispersers (*20*) similar to an increasingly recognized interaction between microbes and pollinators (*21*).

Here, we characterize the availability of ethanol in ripe fruits consumed by chimpanzees at two field sites across the species’ geographical range—at an East African locale inhabited by Eastern chimpanzees (*P. troglodytes schweinfurthii*) and a West African site inhabited by Western chimpanzees (*P.t. verus*). We then estimate daily rates of ethanol ingestion by chimpanzees and show that these rates are comparable to contemporary drinking patterns in modern humans.

## RESULTS

### Feeding-time weighted percent ethanol

**Fig. 1.**
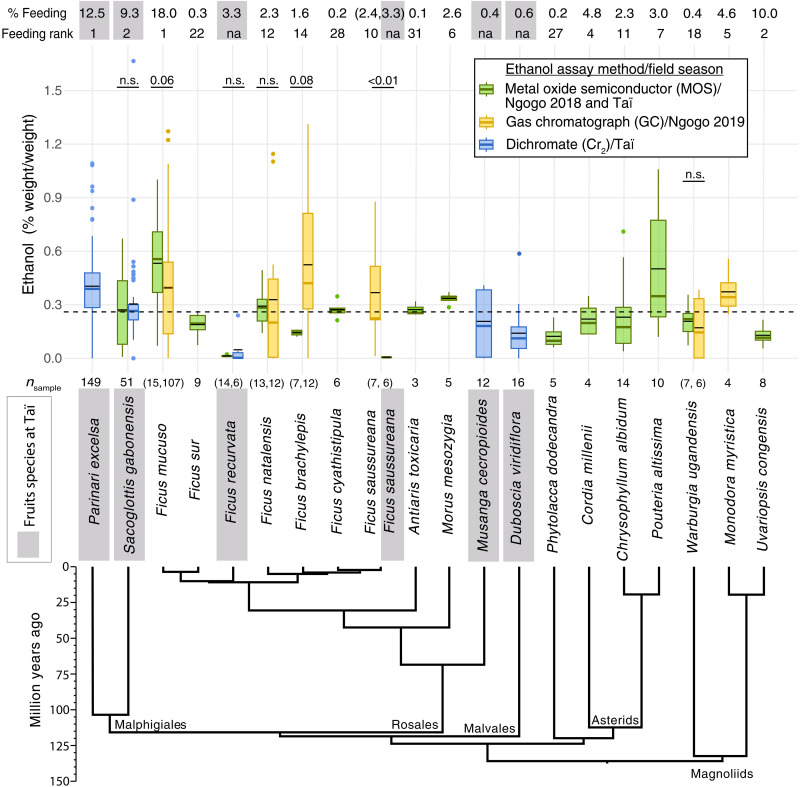
Average ethanol concentrations of fruits at Ngogo and Taï. Taxonomic names of fruit species are arranged according to their evolutionary relationships (*53*), with fruits from Taï highlighted in gray. Boxplots follow standard interquartile range notation and are color-coded by ethanol assay method, as associated with the field seasons specified in the embedded legend. The thicker color-coded line within each box represents the median, and the thin black line represents the mean. Statistical notation above species assayed using two methods indicates the results of a Mann-Whitney *U* test (unpaired for all species except *S. gabonensis*; *V* = 625 and *W* = 1047, 56, 93, 21, 0, and 23, respectively) and are labeled either as n.s. (not significant) or with a *P* value for species at or near the fiducial threshold of 0.05. A dashed horizontal line across the plot indicates the overall pooled species average of ethanol concentrations (0.26%). The top two rows of numbers indicate the annual percentage of time spent feeding on each species by chimpanzees, along with their feeding ranks among all fruits (including figs) at either Ngogo (*22*) or Taï (*23*). Annual feeding percentages for the two *Ficus* species at Taï (representing 3.3% of annual feeding time) were pooled. Sample size (*n*) for each cluster of fruit is indicated above the name of each species.

Ethanol concentrations were estimated for 106 ripe fruit samples belonging to 13 angiosperm species consumed by Eastern chimpanzees at Ngogo in Uganda in 2017–2018 using the metal oxide semiconductor (MOS) method (see Materials and Methods). The linear regression derived from calibration of this instrument yielded an adjusted *R*^2^ (coefficient of determination) of 0.94 (*P* < 0.001) and a mean absolute error (MAE) of 0.07. The taxa assayed correspond to ~65% of the annual time spent feeding on fruit by chimpanzees at this site (Fig. 1) (*22*). Scaling species-level means for all 13 species by the corresponding annual fractional feeding time by Ngogo chimpanzees (*22*) resulted in an average ingested ethanol concentration of 0.35% (weight/weight for all ethanol percentages; see table S1 for calculations). Nearly 60% of this value is derived from fruits of *Ficus mucuso* due to its relatively high ethanol concentrations (0.53%, *n* = 15) and the high annual fraction of feeding time spent on this species (18%). The remaining fraction of ingested ethanol reflects contributions from the eight next most frequently consumed fruit species in the study sample. The second-most consumed fruit species at Ngogo (*Uvariopsis congensis*) composes 10.0% of feeding time, with an average ethanol concentration of 0.13% (*n =* 8).

Ethanol concentrations were separately estimated for 148 ripe fruit samples belonging to six angiosperm species the following year at Ngogo, corresponding to ~41% of the annual feeding time, and including four species sampled the previous year (Fig. 1). These fruits were assayed using the gas chromatograph (GC) method; 11 separate calibration series were used to estimate the ethanol concentrations within 11 corresponding batches of fruit samples, resulting in linear models with adjusted *R*^2^ ranging between 0.65 and 0.92 (0.77 average; all *P* < 0.003) and MAE ranging 0.19 to 0.34 (average 0.29). These species resulted in a weighted average of 0.36%, with *F. mucuso* representing 67% of the value.

Pooling sample data across assay methods and field seasons at Ngogo, with 254 fruits across 15 species corresponding to ~79% of annual feeding time, resulted in a weighted ingested ethanol concentration of 0.32%. The *F. mucuso*, which averaged a pooled concentration of 0.41%, contributed 44% of the weighted value, with the following nine species composing the rest.

Among Western chimpanzees at Taï in Côte d’Ivoire, ethanol concentrations were estimated for ripe fruit samples (*n* = 245) from six angiosperm species, which correspond to 32% of the annual time that Taï chimpanzees spend eating fruit (Fig. 1) (*23*). Of these, 225 samples from five species were analyzed using the dichromate (Cr_2_) method; 17 calibration series were paired with their respective samples, resulting in linear regressions with adjusted *R*^2^ values ranging between 0.95 and 1.00 (all *P* < 0.001) and MAE values ranging over 0.00 to 0.05. Another 17 samples from two species of fig were assayed using the MOS method (11 samples from *Ficus recurvata* and 6 from *Ficus saussureana*), alongside an additional 56 samples of two species assayed using both the Cr_2_ and the MOS methods for cross-validation (51 samples from *Sacoglottis gabonensis* and 5 from *Parinari excelsa*). In all, a series of seven MOS calibrations resulted in linear regressions with adjusted *R*^2^ values ranging 0.98 to 1.00 (all *P* < 0.045) and MAE ranging 0.01 to 0.03. Taï chimpanzees spend more time consuming *Parinari excelsa* than any other food item, which composes 12.5% of total annual feeding time; ethanol concentrations for fruits of this species averaged 0.40% (*n* = 149). The second-most consumed fruit species, *S. gabonensis*, averaged 0.30% ethanol (*n* = 51). The weighted average ethanol concentration among all six species (with two species of *Ficus* pooled as *Ficus* spp.) was 0.31%; 97% of this weighted average is represented solely by *P. excelsa* and *S. gabonensis*. All fruit sample ethanol data can be found in table S2, and calibration standards in table S3.

Phylogenetic relationships did not play a role in explaining ethanol concentrations for any combination of field seasons/methods (table S4). Species identity on its own explained a modest amount of variation in ethanol concentrations for six species collected using Cr_2_ and MOS methods at Taï (*R*^2^_lik_ = 0.26), and 13 species collected using the MOS method at Ngogo in 2018 (*R*^2^_lik_ = 0.24). Species identity did not explain any variation in ethanol values for six species collected using the GC method at Ngogo in 2019 (*R*^2^_lik_ = 0.03).

### Body mass–specific dosage estimates

We estimated fruit ingestion rates across sexes and field sites using values from published literature (see Materials and Methods; Ngogo females: 4.3 kg/day; Ngogo males: 4.5 kg/day: Taï females: 4.6 kg/day; Taï males: 4.8 kg/day) (*24*). Using the weighted ingested ethanol concentration of 0.32% for pooled data at Ngogo and 0.31% for Taï, the corresponding rates of ethanol range from 13.6 to 15.0 g/day for male and female chimpanzees. Body mass–specific estimates of ethanol ingestion are much higher, however, because chimpanzees are smaller than humans. The mass-specific dosage for a 35.9-kg female of the Eastern chimpanzee subspecies corresponds to the equivalent of 0.38 g/kg per day dosage (±0.24 SD), which is the mass equivalent of 2.6 standard drinks (±1.7) daily for a 70-kg human (Table 1).

**Table 1.** Estimated daily body mass–specific ethanol dosage. Fruits compose 71% of the diet of chimpanzees at Ngogo (*22*) and 76% at Taï (*23*); these values were multiplied by the bulk food estimates to calculate the daily mass of fruit consumed. Ethanol concentrations were weighted by estimated percent annual time chimpanzees spend consuming each fruit species (*22*, *23*) (see Materials and Methods), and grams of pure ethanol were derived by multiplying the concentration by the estimated daily mass of fruit consumed. Dosage corresponds to grams of pure ethanol divided by estimated chimpanzee body mass. The equivalent number of standard drinks were calculated by dividing chimpanzee ethanol dosage by 0.14, i.e., the ratio of 10 g of ethanol contained in one standard drink (*57*) over 70-kg human body mass.

Site	Sex	Body mass (kg)*	Bulk food (g)†	Fruit consumed (g)	Ethanol % w/w (± SD)	Ethanol (g) (± SD)	Dosage (g/kg/day) (± SD)	Standard drinks (± SD)
Ngogo	F	35.9	6020	4274	0.32 (0.20)	13.6 (8.7)	0.38 (0.24)	2.6 (1.7)
Ngogo	M	42.0	6333	4496	0.32 (0.20)	14.3 (9.2)	0.34 (0.22)	2.4 (1.5)
Taï	F	41.6	6020	4575	0.31 (0.21)	14.0 (9.6)	0.34 (0.23)	2.4 (1.6)
Taï	M	46.3	6333	4813	0.31 (0.21)	15.0 (10.1)	0.32 (0.22)	2.2 (1.5)
Human reference‡		70.0				10.0	0.14	1.0

* Body masses of chimpanzees based on (*55*) for Ngogo and (*56*) for Taï.

† Bulk food estimates are calculated from (*24*).

‡ A human reference consuming one standard drink (*57*).

## DISCUSSION

Average ethanol concentrations of chimpanzee-consumed fruit, as weighted by the percentage of feeding time that each species is consumed in nature, were consistent across sites (i.e., 0.32% when pooled across two field seasons at Ngogo and 0.31% at Taï). These values are somewhat higher than the mean interspecific concentrations at each site, namely, 0.29% (*n =* 15) and 0.18% (*n =* 6), respectively. Moreover, the most frequently consumed fruit species (on an annual basis) at each site contained some of the highest overall concentrations across the dataset (i.e., 0.41 ± 0.31% for *Ficus musuco* as pooled from two field seasons at Ngogo and 0.40 ± 0.19% for *P. excelsa* at Taï; Fig. 1). Given the large variation in ethanol concentrations associated with these fruit species, including some samples with zero or near-zero ethanol content, chimpanzees could potentially be behaviorally avoiding consumption of fruits with higher concentrations. Conversely, if chimpanzees are attracted to ethanol as we had originally hypothesized, then they could potentially consume fruits with higher concentrations and thereby increase their daily ethanol dosage. All ethanol concentrations are reported here in units of alcohol by mass, but due to the low density of ethanol, conversion to the more familiar units of alcohol by volume would result in ~30% higher concentrations. These overall levels of ethanol are consistent with data from multiple previous studies, which suggest that fruit ethanol content is fairly low and typically <1% (*18*, *25*). In Panama, average ethanol concentrations of ripe pulp averaged 0.5% in the palm *Astrocaryum standleyanum* (*26*, *27*) and averaged 1 to 2% for ripe hogplums (*Spondias mombin*), which were partially consumed and dropped by spider monkeys (*28*). By contrast, ethanol in floral nectar of the bertam palm (*Eugeissona tristis*) consumed by pen-tailed tree shrews (*Ptilocercus lowii*) and slow lorises (*Nycticebus coucang*) in Malaysia averaged 0.6% but ranged as high as 3.8% (*29*). The relevance of all such fruit-ethanol concentrations depends, in part, on the rate and extent of consumption by the frugivore. Given the substantial daily fruit masses consumed by chimpanzees in the literature, the concentrations we report correspond to substantial chronic levels of ethanol intake (i.e., 14 to 15 g/day). The estimated body mass–specific dosages for both female and male chimpanzees are high compared to those typically experienced by modern humans (Table 1).

These results provide the first estimates of daily ethanol intake via frugivory by wild chimpanzees, but we note some important caveats. First, we used a different ethanol assay method during each field season. While this helped ensure a broad assessment of ethanol, it limited comparability of data across field seasons. Future studies would best use at least two methods to assay large samples of each species, as was done here with *S. gabonensis* using a headspace and a reagent-based method. The fuel cell breathalyzer headspace assay method, used originally in (*26*) and more recently in (*30*), may serve as an additional source of validation. The field-portable methods used here can also be compared to laboratory-based approaches such as enzymatic assays and gas chromatography–flame ionization detection (GC-FID), with the goal of better determining pulp-ethanol concentrations in the field. Second, estimates of daily ethanol dosage for chimpanzees (Table 1) were based on body mass and dietary data obtained from prior research. Future comprehensive studies of dietary ethanol ingestion could incorporate year-long observations of individual chimpanzees with information regarding the specific food items these individuals consume. Third, because animals are expected to first consume the most highly desirable fruits, we likely disproportionately sampled the less desirable fruit specimens. If chimpanzees and other animals are attracted to ethanol as originally hypothesized, then this effect would result in conservative estimates for daily ingestion rates. Future studies may artificially exclude frugivores from some crops to evaluate fruit-ethanol values in the absence of animal foragers.

Accurately assaying fruit-ethanol concentrations to test ecological hypotheses is a nontrivial task. Ethanol concentrations were recently estimated for a wide variety of fruit species in Costa Rica, with the conclusion that fruits with mammal-dispersed seeds (as defined by larger fruit size) have higher ethanol concentrations than those that are bird-dispersed (*30*). This study used vapor sampling via a breathalyzer from fruits placed within a plastic bag (for 1 hour), in a method similar to that in reference (*26*) and to the headspace methods used here (MOS and GC). Reference standards of 5 ml of water/ethanol mixtures were used for calibration. However, because fruits ranged from 1.2 to 149 g and were studied whole without extraction of the pulp or removal of the rind, the surface area for ethanol diffusion into the airspace may have varied considerably relative to the reference standards. The surface area and rind thickness of fruit samples in (*30*) were not reported, and resulting ethanol vapor measurements from surrounding headspace may underrepresent internal concentrations of ethanol. Despite this, the overall range of ethanol concentrations reported for wild fruits are broadly consistent with our results, providing further support for ethanol being commonplace in tropical fruits albeit at relatively low values.

Mammalian behavioral responses to ethanol exposure are not well characterized. In some cases, ethanol levels above 1% may deter feeding, as in Egyptian fruit bats (*25*, *31*). Other mammals may, however, be attracted to higher levels. Captive elephants in South Africa preferentially select marula fruits (*Sclerocarya birrea*) with higher sugar concentrations based on the olfactory sensing of associated ethanol (along with ethyl acetate, an ester of acetic acid) within the fruit headspace (*32*). The nectarivorous aye-aye (*Daubentonia madagascariensis*) and slow loris (*N. coucang*) both prefer progressively increasing ethanol concentrations (up to 4 to 5%) in artificial nectar (*33*). Among the great apes, captive chimpanzees (*P. troglodytes*) and orangutans (*Pongo* sp.) voluntarily consume fruit juice containing 10% ethanol (*34*), and wild chimpanzees habitually pilfer human-collected fermented palm sap (*35*).

Chimpanzee foraging and social behavior can be influenced by the presence of ripe fruit crops and by implication of ethanol. Crops of the fig *F. mucuso* at Ngogo attract large groups of chimpanzees (*36*), which in turn results in increased social interactions for both sexes and in social activities such as territorial boundary patrols and hunts (*37*, *38*). Moreover, the initiation of territorial patrols at Ngogo is positively correlated with male party size (*39*) and occurs during periods of high fruit availability and consumption (*40*). Chimpanzee consumption rates of fruit can be variable through the day and may include binge feeding. Ethanol exposure can therefore be correspondingly high—the ingestion of ~75 ripe figs in a single feeding bout would yield 10 g of ethanol, although the behavioral consequences of this dosage are unknown.

Similarly, our estimates for body mass–specific ethanol dosage via frugivory are high (Table 1), but the efficiencies of metabolic clearance pathways in chimpanzees are unknown. Haplorrhine primates (including the great apes) have at least three copies of *alcohol dehydrogenase 1 (ADH1)* that encode the enzyme primarily responsible for ethanol catabolism in the liver, consistent with neofunctionalization (*41*). Also, an *ADH4* mutation shared by humans, chimpanzees, and gorillas encodes an enzyme responsible for first-pass metabolism of ethanol in the esophagus and stomach and resulted in a 40-fold increase in catalytic efficiency of this enzyme (*42*). Catalytic efficiencies for other ADH isozymes involved in ethanol metabolism (or for aldehyde dehydrogenases) have not been characterized for chimpanzees but may have similarly been targets of positive selection given sustained dietary exposure.

These findings also suggest that the occurrence of nontrivial amounts of ethanol within tropical fruits is widespread, and the diversity of methods and timing by which fermentative microbes disperse onto and into fruits are worth considering. Entry into fruit pulp can occur via multiple modes, including vectoring by insects into fissures in the exocarp. Microbes may also be introduced into the pulp by burrowing insect larvae, and within nectaries of female flowers, they may become encapsulated by the developing fruit tissue. Microbes may also enter fallen fruits through microfractures resulting from ground impact, although we found no difference in ethanol concentration between species of fruits typically consumed on the ground (0.27%, *n =* 6 species) versus those consumed in the canopy (0.26%, *n =* 14; Student’s *t* test, *t*_12_ = −0.27, *P =* 0.791). For figs, visitation by fig wasps (Agaonidae) provides a means by which yeasts can enter fruits and proliferate at an early stage of development. Fig wasps enter the infructescence (i.e., the syconium), lay eggs in gall flowers, and pollinate the reproductive flowers while simultaneously inoculating the interior with diverse microbes (*43*). Following eclosion, the next generation of fig wasps (and associated microbiome) then exits the fruit. At Taï, neither fig species contained much ethanol. Twenty fruits of *F. recurvata*, collected the same day and at the same tree, were assayed separately using the Cr_2_ method for 6 samples (resulting in 0.05% ethanol) and the MOS method for 14 samples (0.01%). The other fig, *F. saussureana*, contained 0.01% ethanol (MOS method, *n =* 6). In contrast, six species of fig at Ngogo averaged 0.32% ethanol as pooled across methods, including 0.37% ethanol for seven samples of *F. saussureana* (i.e., the same species at Taï). These observations may reflect a starkly contrasting microbial ecology of fig wasps across these two sites (Fig. 1). Regardless of source, proliferating yeasts are commonplace within fruit pulp (*44*–*46*), but the consequences of microbiome composition and fermentation for the evolution of frugivore-angiosperm mutualisms more generally are not well characterized.

Empirical results presented here suggest that chimpanzee dietary exposure to ethanol can be both chronic and considerable. Moreover, these findings are generalizable to other frugivorous primates and indeed to all tropical vertebrates with a fruit-based diet. Potential roles of ethanol in primate feeding ecology include use of ethanol as a proximate olfactory cue to evaluate fruits suitable for consumption, long-distance sensing of ethanol plumes from fruit for localization, and appetite stimulation increasing rates of fruit ingestion. Ethanol is widespread within fruits of tropical angiosperm species, and the data presented here suggest that its consumption via frugivory is a natural and commonplace phenomenon for frugivores, including human ancestors within Afrotropical forests.

## MATERIALS AND METHODS

### Study sites

In East Africa, fruit-ethanol data were collected at the site of the Ngogo Chimpanzee Project (*47*) (Ngogo; 0°29′55″N, 30°25′30″E; altitude, ~1400 m) in Kibale National Park, Uganda, over two separate field seasons from October 2017 to June 2018 and August to December 2019. To conduct this work, permits were obtained from the Uganda National Council for Science and Technology (permit NS41ES) and the Uganda Wildlife Authority. In West Africa, fruit-ethanol data were collected at the site of the Taï Chimpanzee Project (*48*) (Taï; 5°52′4″N, 7°20′21″W; altitude, ~200 m), Taï National Park, Côte d’Ivoire, from June to September of 2021. To conduct this latter work, a permit was obtained from the Office Ivoirien des Parcs et Réserves of Côte d’Ivoire. Additional data pertaining to chimpanzees used in this study were obtained from previously published studies; the researchers had no direct contact with chimpanzees during the course of this work, and thus, no institutional animal approvals or animal research permits were needed.

Continuous, long-term research on the Ngogo chimpanzees began in 1995, and at the start of this study, the community there was the largest group of chimpanzees ever observed with more than 200 individuals (*47*). During this study, the community split in January 2018 (*49*). The Ngogo chimpanzees are ripe-fruit specialists, spending the highest percentage of annual feeding time on *F. mucuso* (~18%) (*22*), a relatively large fig obtained primarily in the canopy (*36*). The daily minimum and maximum temperatures, collected at the site, averaged 17.0° and 25.1°C, respectively, during the first field season, and 16.8° to 23.0°C during the second field season.

Taï is home to four habituated communities of chimpanzees, each typically composed of 20 to 40 individuals, with the North Group having been studied since 1979 (*50*). Chimpanzees at Taï are followed and monitored daily by field assistants, volunteers, and researchers, and their diet is well-characterized. Taï chimpanzees of the North Group spend the majority of their time (12.5%) feeding on *P. excelsa* and 7.5% of their time on *S. gabonensis* (*23*), both of which are consumed almost exclusively on the ground. The daily minimum and maximum temperatures collected at the site during the study period averaged 24.4° and 28.3°C, respectively.

### Sample collection

Fruit samples were collected opportunistically from crops observed being consumed by chimpanzees. In the case of *P. excelsa*, recent chimpanzee feeding was confirmed for each crop using camera traps (GardePro A3, Hong Kong, China). Substantially damaged or partially eaten fruits were not collected, with the exception of *Musanga cecropioides* at Taï, which could only be collected as freshly broken pieces dropped from the canopy by chimpanzees. For fruit species consumed exclusively in the canopy, samples were only collected if we had witnessed the fruit fall or otherwise inferred that the fruit had recently fallen, e.g., by wet sap on the stem; visibly oxidized and aging fruits or fruits that had been on the ground for an indeterminate period were excluded. Fruits consumed by chimpanzees exclusively on the ground were likewise collected only if they appeared recently fallen, via examination of the portion of the fruit touching the ground; fruits that were moldy, saturated with mud, or embedded in the ground were avoided. Samples were double-bagged, transported to the field laboratory, and then frozen in a −5°C freezer to arrest fermentation until subsequent analysis. Fruit transit times from fruit crop to field lab were minimized and varied depending on distance to camp, averaging ~2 hours.

Estimates of fruit ripeness were determined in the field at both Ngogo and Taï by researchers and field assistants with extensive experience identifying and classifying ripe fruit ingested by chimpanzees. Typically, the individual fruits of the fruit crops we studied developed and ripened more or less synchronously, and the transition in ripeness status of most species was identifiable, signaled by species-characteristic changes sensible to humans, e.g., changes in color, an increase in mass, and becoming softer when palpated. Some fruits among otherwise ripe fruit crops nonetheless occasionally stood out as unripe or underdeveloped and were labeled as such. And at Ngogo, we repeatedly observed chimpanzees briefly eating small crops of crunchy deep-green obviously unripe figs, presumably as a fallback dietary strategy in the absence of higher quality food. All such fruits, classified as unripe, were excluded from this study.

During the first field season at Ngogo, we collected several fruits ingested by chimpanzees, resulting in relatively small samples of each species. During the subsequent field seasons, we prioritized the collection of the aforementioned top fruit species consumed by chimpanzees at their respective sites, namely, *F. musuco* at Ngogo and *P. excelsa* and *S. gabonensis* at Taï, with any other available species sampled haphazardly in smaller quantities. Only species with at least three ripe fruit samples were included in the analysis.

### Fruit ethanol assays

We used three methods to assay fruit ethanol concentrations, developed sequentially during three field seasons (described individually below). In 2017–2018 at Ngogo, we used only a MOS sensor to estimate gaseous ethanol within fruit slurry headspace. This method and the overall experimental approach were based loosely on (*26*). In anticipation of returning to Ngogo in 2019, we successfully piloted another very similar method but instead using a field-portable GC that uses a micro-electro-mechanical system sensor to estimate concentrations of gaseous ethanol within fruit slurry headspace. Both the MOS and the GC methods require careful time-consuming laboratory work, and to streamline logistical constraints in the field, the MOS sensor was not used in 2019. However, the GC instrument proved very sensitive to the tropical humid air (discussed below). Thus, in anticipation of a third field season at Taï, we successfully piloted a different nonheadspace assay method, this time using a chemical assay kit based on dichromate (Cr_2_) as the primary reagent, which changes color in proportion to its reaction to ethanol. To validate these methods, a subset of fruits at Taï was also analyzed using the MOS method. Despite attempts to similarly validate the GC method, this instrument entirely failed to operate at Taï due to the persistently high levels of humidity (>90%), which were substantially less pronounced at Ngogo.

All methods (described in greater detail below) required initial preparation of fruit slurry from defrosted samples. Exocarp and seeds were excluded whenever these were not consumed by chimpanzees. For species of *Ficus*, seeds and exocarp were always included in the assay. Fruits were cut into longitudinal sections, inserted into a 50-ml centrifuge tube and homogenized into a slurry using a battery powered homogenizer (14-mm tip diameter; Tissue-Tearor, Biospec Products, Bartlesville, OK, USA). To convert each method’s raw data outputs to ethanol concentrations, we made calibration standards by serially diluting 70% ethanol with purified water. Calibration standard series were then used to create linear regressions, and the slope and intercept of which were used to predict the ethanol concentration of each associated fruit sample. Negative values were rounded up to zero. Ethanol concentrations were always calculated in units of percent mass, namely, weight over weight (% w/w). For calibration standards, see table S3. All equipment in contact with fruit slurry was sterilized before each use by soaking in 1% bleach solution for 15 min.

### Statistical analyses

To determine whether parametric or nonparametric statistics should be used to compare groups, we first assessed the normality of each group or the normality of the residuals of analyses of variance (ANOVAs). Normality was checked using the Shapiro-Wilk test of normality (“shapiro.test” function in the “stats” package) in R (v4.4.2) (*51*) and by examining the histogram using the “hist” function and the QQ-plot using the “ggqqplot” function. Differences between pairs of groups were then assessed using either a Student’s *t* test (“t.test” function) or the nonparametric Mann-Whitney *U* test (“wilcox.test” function), accordingly; all tests were two-tailed. Differences between multiple groups were assessed using ANOVA (“aov” function) or the nonparametric Kruskal-Wallis test (“kruskal” function in the “agricolae” package).

Phylogenetic effects, on average, species ethanol concentrations were examined using a phylogenetic linear mixed model approach using the “pglmm” function in the “phyr” package (*52*). The species phylogeny was obtained using the “phylo.maker” function of the “V.PhyloMaker2” package in R (*53*). Two models of ethanol concentrations were compared, one a linear mixed model incorporating only species identity as a random effect and another that added phylogenetic relationships as another random effect. Additional coefficient of determination estimates associated with each model were made using the “R2” function in the “rr2” package (*54*) using the *R*^2^ value associated with maximum likelihood (*R*^2^_lik_) for comparison. This paired model approach was repeated across datasets with combinations of each method and field season separately and pooled.

### MOS sensor method

This method used a vapor alcohol sensor (TGS2620, Figaro USA Inc., Arlington Heights, IL, USA; ETH-BTA, Vernier Software and Technology, Beaverton, OR, USA) connected to a touchscreen interface (LabQuest 2, Vernier Software and Technology) and datalogger software (LoggerPro 3.15, Vernier Software and Technology) to sample alcohol concentrations within the headspace above fruit slurry. The electrical conductivity of the sensing chip increases when alcohol vapors contact the internal MOS layer. The sensing chip is recommended for “alcohol testers” and “organic vapor detectors/alarms” by the manufacturer (TGS2620, Figaro USA), and therefore although the instrument is “very sensitive to ethanol vapor”, it can also respond to other organic volatile compounds (ETH-BTA, Vernier Software and Technology). Fruit samples analyzed using this method were first diluted 1:1 with purified water to facilitate homogenization. Exceptions to this dilution factor included two samples of *Phytolacca dodecandra* and one sample of *U. congensis*, which were analyzed undiluted during early sampling at Ngogo and which were sufficiently watery to not require dilution. At Taï, seven samples of *S. gabonensis* were diluted threefold and one sample of *P. excelsa* fourfold to match the dilution factor of the accompanying Cr_2_ method during cross-validation (see below). Five grams of fruit slurry (or calibration standard) was pipetted into a 50-ml self-standing centrifuge tube, which was then sealed with a nonporous oven bag plastic to allow gas equilibration between sample and headspace. To record output voltage, we made a cut in the plastic and inserted the alcohol sensor 1 cm above the slurry until the voltage remained constant for 20 s.

For fruits assayed using this method at Ngogo in 2018, headspace was equilibrated for 15 min before sample collection. The instrument was precalibrated in the laboratory before arrival at Ngogo by pooling multiple serial dilutions ranging from 1.6 to 0.0125% ethanol, across *n =* 27 standards. Because this method can be sensitive to temperature, we documented the internal temperature of each calibration standard in the laboratory using a stainless steel temperature probe (Vernier Software and Technology); values averaged 22.9°C (range 22.0° to 23.8°C). While at Ngogo, we likewise recorded all fruit sample temperatures, cooling the sample tubes in ice water or warming them using body heat as needed, which resulted in an average value of 22.4°C (range 21.3° to 23.1°C). At Taï in 2021, headspace was equilibrated for up to 8 hours, with calibration standards being analyzed alongside samples and thus eliminating the need to keep track of temperature. Rather, four series of calibration standards were prepared by serial dilution, composed of 0.8, 0.4, 0.2, and 0.1% ethanol and were assayed alongside four series of fruit samples representing *n* = 71 fruits; another three calibration series associated with a total of 11 fruits did not contain the 0.8% standard. The factory calibration of this instrument defaults to a power regression, and we therefore log-transformed the known ethanol concentrations of our calibration standards to obtain a linear regression for the series. Resulting fruit ethanol values were then exponentially transformed and multiplied by the dilution factor.

To assess the effect of headspace equilibration time with fruit slurry on ethanol concentration measurements using this method, we separately assayed samples from the same pool of fruit slurry over increments of 0.25, 0.5, 1, 2, and 4 hours using three *S. gabonensis* fruits at Taï. No significant differences in ethanol concentrations were observed within the headspace of these samples (repeated measures ANOVA, *F*_4,8_ = 1.7, *P* = 0.245). Fruit-ethanol concentrations over time were consistent and differed from the mean of each sample, on average, by 8%. Differences over 4- and 8-hour increments using another three samples from the same species were nearly significant (Student’s paired *t* test, *t*_2_ = 3.9, *P* = 0.059), with all three decreasing by an average of 8% of the 4-hour value (fig. S1). Using these data, we also estimated the MAE associated with repeated sampling of the same fruit slurry using this method, which averaged 0.004% (ranging 0.001 to 0.008%) relative to an average ethanol concentration of 0.10% (ranging 0.02 to 0.18%).

### Portable GC method

A portable GC (SeaPORT Mini-GC, Seacoast Science Inc., Carlsbad, CA, USA) was used to measure ethanol concentrations of headspace over fruit slurry at Ngogo in 2019. This device separates constituent gases by molecular weight and exposes them to a micro-electro-mechanical system sensor that produces a corresponding voltage output over time. Thus, in principle, other short chain alcohols such as methanol and isopropyl alcohol could inflate an ethanol peak, although these other alcohols are not typically major products of fermentation. The GC interfaced with the same software as the aforementioned MOS method (namely, Logger Pro 3.15); ambient air was used as a carrier gas. Samples were prepared by evenly applying 1.5 g of fruit slurry to a 30-mm petri dish, covered with nonporous plastic oven bag material and rubber bands, resulting in 3.5 to 4 ml of headspace. We then used a gastight syringe (1001 LT SYR with Kel-F hub point style 2 needle, Hamilton Company, Reno, NV, USA) to pierce the plastic covering of the petri dish to extract 1 ml of headspace for injection into the GC. All headspace measurements were collected at a continuous column temperature of 40°C and pressure of 7 kPa. Samples were not diluted during this field season, as all fruit species chosen for analysis contained sufficient water content to not require it.

To interpret the ensuing GC signal, we used the “Integral” tool in Logger Pro to estimate the area under each peak (in units of mV·min). On the basis of observations of ethanol calibration standards obtained from the instrument in the field, the initiation of a fruit sample ethanol peak was expected to take place 50 to 54 s after sample injection. To reduce error due to a delayed return to baseline after a signal peak, we estimated the time of end of each peak as when the signal decreased to 1.2 mV above baseline.

Two series of calibration standards—composed of 2.4, 1.2, 0.6, 0.3, and 0.15% ethanol—were analyzed, one immediately before and one after each series of fruit samples, using separately prepared standards. Because of this instrument’s sensitivity to water and its use of ambient air as a carrier gas, samples could only be analyzed on the GC during times when the ambient relative humidity at Ngogo fell below ~60% during mid-day. Because of these logistical and timing constraints, samples were prepared the night before and stored in a cool insulated place, with resulting equilibration times ranging from 17 to 24 hours (20-hour average). Samples that could not be analyzed within a day were discarded. To prevent fermentation during the equilibration process, all fruits were frozen in a −5°C freezer before sample preparation, and all equipment was sterilized using 1% bleach solution.

### Dichromate (Cr_2_) reduction method

The dichromate method used two reagent kits, one for precipitating saccharides in a liquid sample (DRSK-500, BioAssay Systems, Hayward, CA, USA) and the other for colorimetric estimation of ethanol based on a reduction reaction with dichromate, whose formula is optimized to reduce “interference by (nonethanol) substances in the raw samples” (DIET-500, BioAssay Systems). While the kits were formulated for samples such as “wine, beer, culture media… etc.”, they have not been validated for assaying fruit ethanol. The kit recommends deproteinating protein-rich samples, which we did not do because fruits are not typically considered to be protein-rich, samples were diluted at least twofold before assay, and the solid mass of the fruit was centrifuged out before assay, which would naturally filter out most proteins from the assay supernatant.

Each fruit sample was homogenized after dilution with 1:1 purified water, weighed to 1.5 g within a 1.5-ml centrifuge tube, and centrifuged for 15 min (10K Mini Centrifuge, Qor Labs) to obtain 300 μl of supernatant. If under 300 μl was produced, slurries were rediluted as needed, up to a ratio of 1:4. We then added saccharide-precipitating reagents to both the fruit samples and the calibration standards, waited 15 min for incubation, centrifuged for 5 min, and extracted 400 μl of the resulting supernatant. To this supernatant, we added the assay start reagents sequentially, incubated for 30 min, and then added the stop reagent. We then used a field portable cuvette reader (GDX-SVISPL, Vernier Software and Technology) and associated software (Spectral Analysis, version 4.8.4-1356, Vernier Software and Technology) to measure color absorbance at 580 nm. To make the resulting values comparable to the previously described headspace methods, which estimate the ethanol concentration of the whole fruit rather than just of the supernatant, we also determined the dry mass of the remaining centrifuged fruit pellet. Pellets were put in weigh boats, air-dried in the sun, and placed in plastic bags with large volumes of silica gel. Desiccation time in silica gel bags was determined by repeated weighing of the pulp until it no longer decreased in mass (~72 hours). Dry mass was divided by total centrifuge mass and multiplied by the dilution factor to obtain the dry mass percent of the fruit.

Calibration standard cuvettes were read before and after each series of fruit samples, with each series of standards composed of a serial dilution of 0.8, 0.4, 0.2, and 0.1% ethanol. Bookending the sample assay with calibration standards was necessary because the cuvette reader contains a lamp that is sensitive to changes in temperature and humidity over time, which at times fluctuated substantially at Taï. Ethanol concentrations of fruit samples were determined by multiplying the absorbance by the slope of the calibration series regression plus the intercept. These latter values were then multiplied by the dilution factor and by one minus the dry mass.

To estimate the precision of this method and the variation associated with assaying different segments of the same fruit sample, we cut three fruit samples of *P. excelsa* into thirds and assayed each segment in triplicate, resulting in nine sets of triplicates. Repeated assays of the nine pools of slurry collected in triplicate resulted in a MAE of 0.02% ethanol, relative to an overall mean value of 0.17% (ranging 0.01 to 0.04% across the nine samples). To separately estimate error associated with different parts of the fruit, three longitudinal segments collected from each of three *P. excelsa* fruits yielded MAE values of 0.01 to 0.02%, relative to the same overall mean value of 0.17% (fig. S2).

### Cross-validation of Cr_2_ and MOS ethanol assay methods

To evaluate the consistency of ethanol concentrations as determined by the Cr_2_ and MOS methods, 51 samples of *S. gabonensis* and 5 samples of *P. excelsa* were assayed simultaneously using both methods at Taï (the GC method failed to operate and was not cross-validated). For *S. gabonensis*, the differences in the average concentrations of the MOS and Cr_2_ groups were not significant, averaging 0.27 and 0.30%, respectively (paired Mann-Whitney *U* test, *V* = 625, *P* = 0.725), and for *P. excelsa*, these differences were greater than twofold, and nearly statistically significant (averaging 0.19 and 0.46%, respectively; paired Mann-Whitney *U* test, *V =* 0, *P* = 0.063; fig. S3). A linear model of *S. gabonensis* methods yielded an adjusted *R*^2^ of 0.04 (*P =* 0.18); however, with one extreme outlier among the Cr_2_ group removed (Sg31, identified via the interquartile range method), this value increased to 0.22 (*P* = 0.10); a linear model for *P. excelsa* was not significant (fig. S4). Thus, despite nearly identical group means, the distribution of the data is less well correlated than might be expected (*R*^2^ = 0.22; fig. S4). To determine whether these differences are within the expected maximum error range of the combined methods, we first calculated the maximum variation of the known versus the predicted ethanol concentrations of the associated calibration standards of each method and added them together, resulting in an average of 0.09% (or 0.07, 0.13, 0.06, and 0.09% corresponding to calibration standards binned into 0.1, 0.2, 0.4, and 0.8%, respectively). To these, we added the maximum variation in estimated ethanol concentrations from repeatedly sampling from the same pool of fruit slurry (figs. S1 and S2), which contributed another 0.05% of error, totaling 0.14% maximum error. We then averaged the estimated fruit ethanol values from both methods, assuming them both to be true, and calculated the absolute difference from this average for each value. Forty-nine of the 51 *S. gabonensis* fruits were under 0.12% error, averaging 0.04%, well under the maximum error range of 0.14%. The two erroneous fruit samples, Sg31 and Sg45, resulted in notably higher concentrations via the Cr_2_ method than the MOS method. However, the removal of these outliers has a minimal effect on the overall results, and since no such protocol exists for the remaining fruit species, we assumed that the Cr_2_ method was correct. It is not possible to similarly analyze the maximum error associated with the only *P. excelsa* cross-validation calibration series. However, when compared to the error threshold associated with *S. gabonensis* calibration standards, the error of all the *P. excelsa* samples was within limits. The observed differences among these method results may in part derive from the different physical approaches used to measure ethanol. The Cr_2_ method relies on optically sensing changes in color due to a chemical change within a liquid medium on the one hand, and the MOS method detects changes in the electrical conductivity of a semiconductor material in response to contact with ethanol vapors on the other. Each method has several other steps in addition to these, each of which may introduce variability that is unique to the method. Assay estimates may also be influenced by between- and within-species differences in fruit pulp biochemistry and tissue structure, with possibly variable effects on each method. Without further methods, validation individual false-positive values remain possible; however, they are unlikely to be systematic, given that one or more calibration series are associated with samples ranging in value from near-zero to the average ethanol value for each species.

### Feeding-time weighted body mass–specific dosage estimates

We obtained weighted averages of ethanol concentrations of fruit species at each field site by scaling their concentration to the reported annual percentage feeding time of that species for Ngogo (*22*, *23*) for Taï based, on averages, from long-term data collected over a 15- and a 25-year period at each site, respectively. These annualized fruit-feeding times may not necessarily be proportional to the annual mass of consumed fruit mass, as feeding rates associated with individual fruit species can be highly variable. Nonetheless, at Ngogo, we collected data for 13 species, which collectively represent ~46% of annual feeding time spent by chimpanzees across all dietary categories. We divided the percentage of feeding time associated with each species by the total percent and then multiplied this value by the average ethanol concentration of that species to determine its contribution to the total estimate. These values were then summed to produce a feeding percent–weighted average.

To convert the percent ethanol to grams ethanol, an estimate of the total daily food mass consumed by chimpanzees is needed. Comprehensive data on daily food mass ingested by wild chimpanzees are scarce and require year-long focal following of multiple individuals, along with careful observation of feeding rates on individual fruit species. The only such published study was conducted at a field site 10 km from Ngogo (i.e., Kanyawara), where female and male chimpanzees were found to consume 6.0 and 6.3 kg of food (including fruit) each day, respectively (*24*). Here, we apply these data to estimate consumption by chimpanzees at Ngogo and Taï, respectively, and note that this feeding behavior may vary from site to site due to differing rates of caloric expenditure and the overall caloric density of available food. Ngogo and Taï chimpanzees spend comparable amounts of time feeding on fruit (71% versus 76%) (*22*, *23*), and these proportions were used to estimate total fruit mass consumed daily at the two sites. These values were multiplied by the weighted ethanol percentages to estimate ethanol dosage in grams for males and females across both sites.

To further estimate body mass–specific ethanol dosage between individuals of different body sizes, we divided grams of pure ethanol by the estimated body masses (kg) of wild female and male chimpanzees of the two subspecies in this study, *P. t. schweinfurthii* and *P. t. verus*. Body masses were estimated from seven adult females and six adult males of *P. t. schweinfurthii* (*55*) and from one male and three females for *P. t. verus* (*56*). Resulting dosages, in units of grams ethanol per kilogram body mass per day of feeding (g/kg per day), were then compared to that of a single standard drink consumed by a 70-kg human. The most commonly used value for a standard drink internationally is 10 g of pure ethanol (*57*). Chimpanzee dosages in grams per kilogram are then divided by the resulting human value of 0.14 g/kg per standard drink to estimate the daily equivalent of standard drinks consumed by chimpanzees.
