# Supplementary material for: Ethanol ingestion via frugivory in wild chimpanzees
Source: Sci Adv. 2025 Sep 17;11(38):eadw1665. doi: 10.1126/sciadv.adw1665 (PMC12442844; doi:10.1126/sciadv.adw1665)
Supplement: Supplementary file 1 — Figs. S1 to S4 Legends for tables S1 to S7 [file sciadv.adw1665_sm.pdf]

Science Advances
AAAS

Supplementary Materials for

Ethanol ingestion via frugivory in wild chimpanzees

Aleksey Maro *et al.*

Corresponding author: Aleksey Maro, alekseymaro@berkeley.edu

Sci. Adv. **11**, eadw1665 (2025)
DOI: 10.1126/sciadv.adw1665

The PDF file includes:

Figs. S1 to S4
Legends for tables S1 to S7

Other Supplementary Material for this manuscript includes the following:

Tables S1 to S7

Fig. S1. Headspace equilibration timeplot for the MOS assay method.

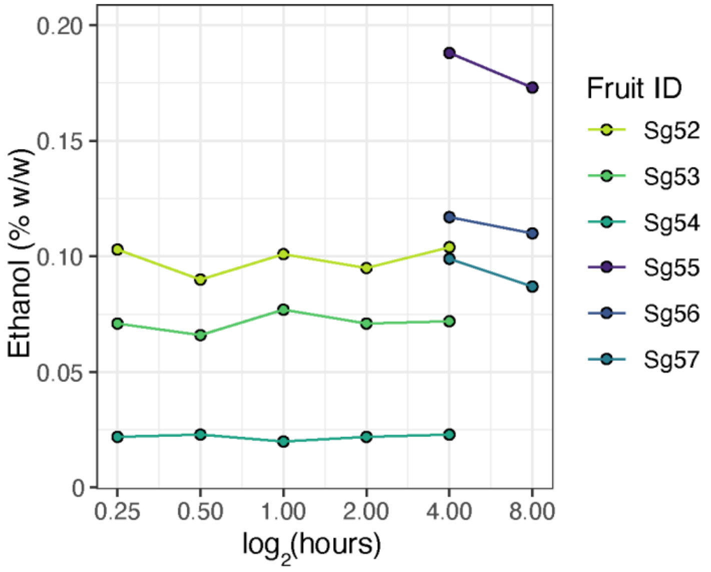

Ethanol concentrations for fruit slurry samples of *Sacoglottis gabonensis* at Taï, collected at incremental headspace equilibration time intervals (time along the *x*-axis is log base$_2$-transformed) using the MOS assay method. Samples each weighed 5 g and were spread out within a surface area of ~28 mm^2 inside a 50 mL self-standing centrifuge tube. Each time point for each individual fruit was prepared separately from the same pool of slurry. No significant statistical differences were observed in ethanol concentrations (see **Methods**). Data used to make this plot can be found in **table S5**. These ethanol concentrations were not corrected for the dilution factor implemented when assaying the fruit and are thus not homologous with the other data sets reported, but statistical results are independent of this linear correction. The mean absolute error (MAE) associated with repeatedly sampling of the same fruit slurry averaged 0.004% (ranging 0.001 to 0.008%) relative to an average ethanol concentration of 0.10% (ranging 0.02-0.18%).

Fig. S2. Error plot for the dichromate (Cr2) assay method.

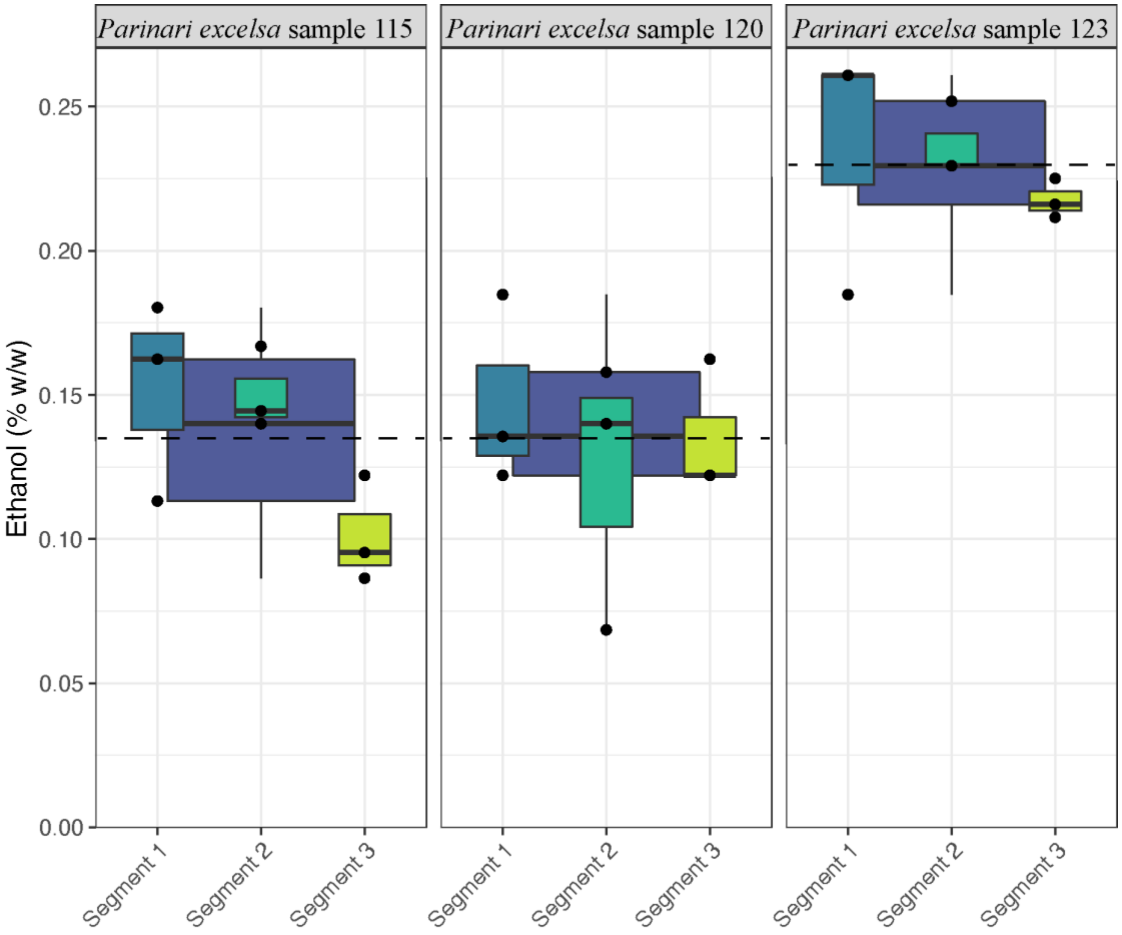

Error plot of ethanol concentrations of three *Parinari excelsa* fruit samples from Taï (2021), collected using the dichromate (Cr2) method. Smaller boxes represent ethanol concentration measurements from three distinct but equal-sized longitudinal segments from each fruit. Larger dark blue boxes in the background represent overall error for a total of nine assays conducted on each fruit (i.e., three assays per segment). Solid lines within each box represent the median and the dashed lines represent the average across all nine samples. The mean absolute error (MAE) from assaying different segments belonging to the same fruit corresponded to 0.01% ethanol, relative to an overall mean value of 0.17%. Segments were not significantly different in ethanol concentration within any of the three samples (One-way ANOVA, $F_{2,6}$=0.4–4.3, *P*=0.070, 0.699, 0.629, respectively). The MAE associated with each of the nine resulting pools of fruit slurry analyzed separately (i.e., the smaller boxes in front of the dark blue larger boxes above, each assayed in triplicate) was 0.02%, relative to a mean value of 0.17%. Thus the overall MAE for each sample, representing the combined error associated with choosing a random segment and replicates for each fruit sample (corresponding to the larger dark blue boxes), was 0.03%. Data used to make this plot are in **table S6**. Ethanol concentrations in this figure were not corrected for either the fruit's dry mass percentage or the dilution factor, but such corrections do not affect the error percentages presented here.

Fig. S3. Cross-validation boxplots for the Cr2 and MOS assay methods.

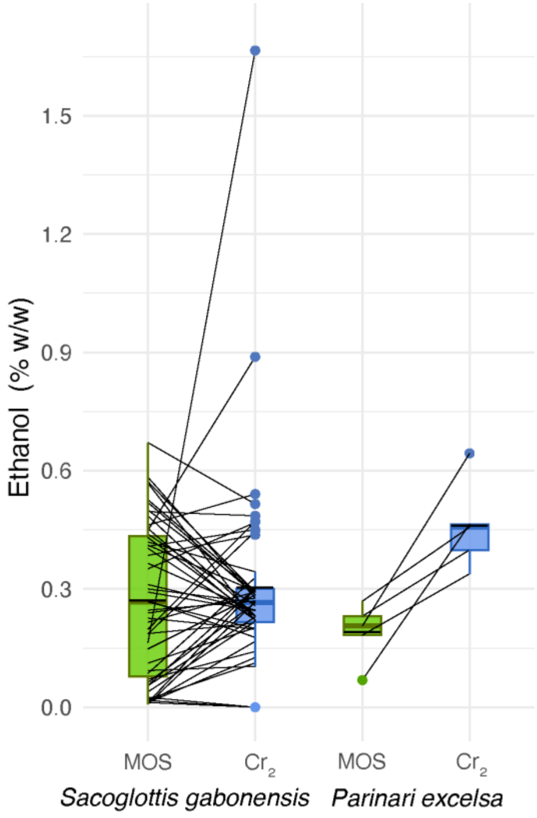

Ethanol concentrations collected using the MOS and the Cr_2 methods for 51 *Sacoglottis gabonensis* and 5 *Parinari excelsa* fruit samples. Thin lines connect values associated with individual samples across boxplots. A thicker green or blue line within each box represents the median value, and a thin black line represents the mean. Data used to make this are in **table S7**.

Fig. S4. Cross-validation regressions for the Cr2 and MOS assay methods.

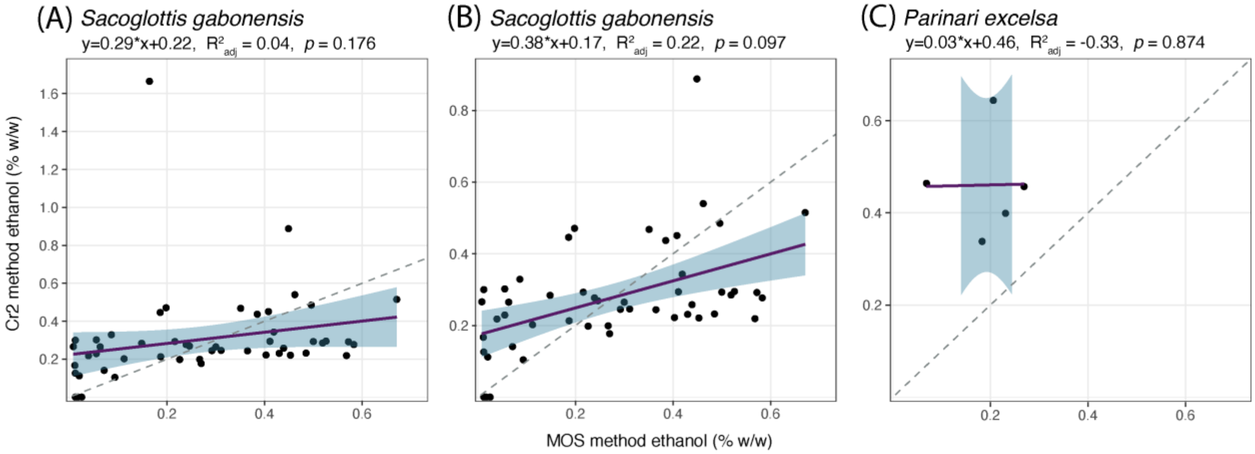

Cross-validation plots for two methods of ethanol assay (the metal oxide semiconductor (MOS) method and the dichromate (Cr2) method), using two species of fruit at Taï. Each plot contains a linear regression with 95% confidence intervals, and the slope, adjusted R^2, and *P*-values indicated above each chart. A perfect correlation (i.e., slope of 1) is indicated by a gray dashed line. **(A)** A plot for 51 samples of *Sacoglottis gabonensis*. **(B)** As in **(A)** but with the left uppermost outlier removed. **(C)** A plot for 5 samples of *Parinari excelsa* fruit. Data used to make these plots are in **table S7**.

Table S1
Fruit ethanol concentrations averaged by species, with % ethanol contribution from each species weighted by chimpanzee annual fractional feeding times, as pooled into sub-tables by field season and method. The column *Species* indicates the genus and species of the fruit, followed, where applicable, by alternate taxonomic names and/or the year(s) that samples were collected. The column *Assay method(s)* indicates the method of ethanol assay: MOS=metal oxide semiconductor method, GC=gas chromatography method, Cr2=dichromate assay kit method. The columns *% feeding Watts et al. (2012a)* for tables associated with Ngogo, and *% feeding Gone Bi & Wittig (2019) North group* for tables associated with Taï, list the fraction of time chimpanzees spend consuming fruit based on references (*22*) and (*23*), respectively. The column *% ethanol contribution* indicates the weighted contribution of percent ethanol by each fruit species, obtained by multiplying the ethanol percent of each species by the ratio of that species to total *feeding %*. This latter column is summed at the bottom of each table for an overall annual feeding-time weighted percent ethanol concentration, as reported in the main text.

Table S2

Raw data associated with all fruit samples presented in **Fig. 1**. The column *Field season* refers to the site and year of one of three data collection periods. The column *Method* indicates the method of ethanol assay: MOS=metal oxide semiconductor method, GC=gas chromatography method, Cr2=dichromate assay kit method. The column *Species* indicates the genus and species of the fruit, followed, where applicable, by alternate taxonomic names. The column *Fruit ID* ascribes a unique sample identification based on the first letter of the genus and species followed by a unique number. The column *Calibration ID* indicates the calibration series that was analyzed alongside each fruit sample (named after the range of Fruit ID samples associated with that calibration series), each of which is listed in **table S3**. For the Ngogo 2018 field season, the Calibration ID is entitled Ngogo2018 because calibration series were collected in the laboratory ahead of the field season. The column *Method signal* corresponds to the raw voltage output for samples analyzed using the MOS method; for the GC method, the method signal is an area under voltage over time, in units of millivolt-minutes (i.e., mV x min); for the Cr2 method, the method signal is the unitless colorimetric absorbance at 580 nm. The columns *Calibration regression intercept* and *Calibration regression slope* refer to the intercept and slope of the linear regression of the calibration series associated with each sample. The column *Dilution factor* indicates whether the extent to which the fruit slurry was diluted with water, in units of mass by mass; a dilution factor of 1 indicates no dilution, a factor of 2 indicates dilution by one-half, etc. The column *Dry mass (Cr2 method)* applies only to the Cr2 method, is reported in decimal form, and is necessary to calculate an accurate ethanol concentration using this method. The column *Ethanol (uncorrected negative values)* is calculated differently for each method; for the MOS method, ethanol concentration is calculated as:

$$ethanol\ \%_{MOS} = e^{(calibration\ slope\ \mathrm{x}\ method\ signal - calibration\ intercept)}\ \mathrm{x}\ dilution\ factor$$

For the GC method, ethanol concentration is calculated as:

$$ethanol\ \%_{GC} = (calibration\ slope\ \mathrm{x}\ method\ signal - calibration\ intercept)\ \mathrm{x}\ dilution\ factor$$

For the Cr2 method, ethanol is calculated as:

$$ethanol\ \%_{Cr2} = (calibration\ slope \text{ x } method\ signal - calibration\ intercept) \text{ x } dilution\ factor \text{ x } (1 - dry\ mass)$$

The final column, *Ethanol (% w/w)* is the same as the previous column, but with all negative values rounded up to zero.

Table S3

Raw data associated with all calibration standards used in this study. The column titled *Ethanol standard (% w/w)* indicates the known ethanol concentration of each standard (series of which were prepared by serial dilution), expressed as a percentage of mass ethanol over total mass. See **table S2** for descriptions of the remaining columns.

Table S4

A generalized linear mixed model (GLMM) compared to a phylogenetic generalized linear mixed model (PGLMM), with data pooled by field season and method (separated by grey highlighting and bolded lines). The column titled *Method/season* refers to one or more methods (MOS, metal oxide semiconductor method; GC, gas chromatography method; Cr2=dichromate assay kit method), and the site and year of one of three data collection periods (i.e., field seasons). The column titled *Model* indicates whether the model was a phylogenetic or a non-phylogenetic generalized linear mixed model (PGLMM or GLMM), and the column *R formula* indicates the corresponding formula as it would be entered in R. The columns *n samples* and *n species* refer to total number of fruit samples and the total number of species to which they belong, respectively. The column R^2_{lik} refers to the coefficient of determination estimate associated with each model, typically interpreted as the percentage of variation explained in the dependent variable by the independent variables. The columns *Random effect species*, *Random effect phylogenetic*, and *Random effect residual* indicate the relative degree to which the level of species categories, higher phylogenetic relationships, and individual samples contribute to model fit. The columns titled *Intercept* and *Intercept p-value* indicate the mean ethanol concentration and its statistical significance following corrections associated with the aforementioned random effects.

Table S5

Data associated with **fig. S1**. The columns *Field season*, *Species*, *Fruit ID*, *Calibration series*, *Intercept*, *Slope*, and *Ethanol (% w/w)* are defined as in **table S2**. The column *Equilibration time (hr)* refers to the number of hours of equilibration between the fruit slurry and its headspace prior to sample collection. The column *MOS signal* refers to the raw voltage signal associated with the metal oxide semiconductor (MOS) method.

Table S6

Data associated with **fig. S2** and **fig. S3**. The columns *Species, Fruit ID,* and *Calibration series* are defined as in **table S2**, and the columns titled *Ethanol Cr2* and *Ethanol MOS* refer to ethanol concentrations derived using the dichromate assay method (Cr2) and the metal oxide semiconductor (MOS) method, respectively.

Table S7

Data associated with **fig. S4**. The column titled *Fruit ID* is as in **table S2,** and refers to three fruits belonging to the species *Parinari excelsa*. The column *Segment* refers to one of three longitudinal segments of an individual fruit specimen, and the column *Replicate* refers to one of three samples of slurry taken from the same segment. The column *Cr2 signal* indicates the raw colorimetric absorbance of the sample at 580 nm, collected after using the dichromate reagent kit. The columns *Calibration series*, *Intercept*, and *Slope* are the same as in **table S2**, and were used to calculate values in the column *Ethanol (% w/w)*.
